# Acceptability and Preliminary Feasibility of an Internet/CD-ROM-Based Education and Decision Program for Early-Stage Prostate Cancer Patients: Randomized Pilot Study

**DOI:** 10.2196/jmir.1891

**Published:** 2012-01-13

**Authors:** Michael A Diefenbach, Nihal E Mohamed, Brian P Butz, Natan Bar-Chama, Richard Stock, Jamie Cesaretti, Waleed Hassan, David Samadi, Simon J Hall

**Affiliations:** ^1^Mount Sinai School of MedicineDepartments of Urology & Oncological SciencesMount Sinai School of MedicineNew York, NYUnited States; ^2^Departments of Urology & Oncological SciencesMount Sinai School of MedicineNew York, NYUnited States; ^3^College of EngineeringTemple UniversityPhiladelphia, PAUnited States; ^4^Department of UrologyMount Sinai School of MedicineNew York, NYUnited States; ^5^Department of Radiation OncologyMount Sinai School of MedicineNew York, NYUnited States; ^6^Florida Radiation Oncology GroupJacksonville, FLUnited States; ^7^Department of UrologyTawam HospitalAl AinUnited Arab Emirates

**Keywords:** Multimedia, software, prostate cancer, patient education, treatment decision making, treatment, decision making

## Abstract

**Background:**

Prostate cancer is the most common cancer affecting men in the United States. Management options for localized disease exist, yet an evidence-based criterion standard for treatment still has to emerge. Although 5-year survival rates approach 98%, all treatment options carry the possibility for significant side effects, such as erectile dysfunction and urinary incontinence. It is therefore recommended that patients be actively involved in the treatment decision process. We have developed an Internet/CD-ROM-based multimedia Prostate Interactive Educational System (PIES) to enhance patients’ treatment decision making. PIES virtually mirrors a health center to provide patients with information about prostate cancer and its treatment through an intuitive interface, using videos, animations, graphics, and texts.

**Objectives:**

(1) To examine the acceptability and feasibility of the PIES intervention and to report preliminary outcomes of the program in a pilot trial among patients with a new prostate cancer diagnosis, and (2) to explore the potential impact of tailoring PIES treatment information to participants’ information-seeking styles on study outcomes.

**Methods:**

Participants (n = 72) were patients with newly diagnosed localized prostate cancer who had not made a treatment decision. Patients were randomly assigned to 3 experimental conditions: (1) control condition (providing information through standard National Cancer Institute brochures; 26%), and PIES (2) with tailoring (43%) and (3) without tailoring to a patient’s information-seeking style (31%). Questionnaires were administrated before (t1) and immediately after the intervention (t2). Measurements include evaluation and acceptability of the PIES intervention, monitoring/blunting information-seeking style, psychological distress, and decision-related variables (eg, decisional confidence, feeling informed about prostate cancer and treatment, and treatment preference).

**Results:**

The PIES program was well accepted by patients and did not interfere with the clinical routine. About 79% of eligible patients (72/91) completed the pre- and post-PIES intervention assessments. Patients in the PIES groups compared with those in the control condition were significantly more likely to report higher levels of confidence in their treatment choices, higher levels of helpfulness of the information they received in making a treatment decision, and that the information they received was emotionally reassuring. Patients in the PIES groups compared with those in the control condition were significantly less likely to need more information about treatment options, were less anxious about their treatment choices, and thought the information they received was clear (*P* < .05). Tailoring PIES information to information-seeking style was not related to decision-making variables.

**Conclusions:**

This pilot study confirms that the implementation of PIES within a clinical practice is feasible and acceptable to patients with a recent diagnosis of prostate cancer. PIES improved key decision-making process variables and reduced the emotional impact of a difficult medical decision.

## Introduction

Prostate cancer is the most common cancer and the second leading cause of cancer-related deaths among American men [1]. In 2011, it is estimated that approximately 217,730 men will have a prostate cancer diagnosis and approximately 32,050 men will die of the disease [1]. Early-stage prostate cancer patients (ie, who present with a tumor that is confined to the prostate, and who have no regional lymph node or distant metastasis, or T1–2N0M0 [2,3]) can choose between several treatment options—surgery (ie, prostatectomy) and radiation therapy (ie, external radiation, brachytherapy, or CyberKnife robotic radiosurgery)—or active surveillance. Although both surgical and radiotherapy approaches have excellent cancer control, each treatment option is characterized by a distinctive pattern of potentially long-lasting urinary, bowel, and sexual dysfunction [3]. In the absence of an evidence-based standard for therapy, it is important for patients to understand how different treatment options will influence their immediate and long-term quality of life. Arriving at a treatment decision can be quite challenging for patients. Most patients are highly distressed after a cancer diagnosis, yet they are required to absorb a large amount of medical information that is often presented in language fraught with medical and probabilistic terms [4-6], and they have to resolve often contradictory medical opinions from consulting physicians of different medical subspecialties. Making a treatment decision under these circumstances is difficult and may lead to emotional distress and subsequent decisional regret, especially if the chosen treatment and its side effects decrease the patients’ quality of life [7].

Electronic and traditional print-based education decision-making materials have been used for patients with localized prostate cancer to enhance treatment decision making. Both types of materials have been shown to improve disease-specific knowledge and to facilitate decision making [8]. Yet few comprehensive Web-based resources are available for prostate cancer patients that combine unbiased treatment information culled from the existing literature, with physicians answering frequently asked questions and testimonials from prostate cancer survivors (eg, [9-11]). We present results from a small pilot study that examined acceptability, feasibility, and preliminary outcomes of a state-of-the-art multimedia intervention, Prostate Interactive Educational System (PIES) [11] designed to educate patients about their treatment options and to facilitate their treatment decision process.

### PIES Description

PIES is designed to present disease- and treatment-relevant information through a variety of electronic media (ie, text, graphics, video clips, and animation) and self-navigational aids, and is a truly innovative, state-of-the-science multimedia preparatory aid for prostate cancer patients (see Multimedia Appendix, Multimedia Appendix 2, Multimedia Appendix 3, and Multimedia Appendix 4). Conceptually, PIES serves as a virtual health center that patients visit to obtain prostate cancer-relevant information. On entering the system, patients are greeted by a health educator who gives them an overview of PIES and its contents (ie, physicians’ offices, library, and support group room). Physicians are represented by videos of actual doctors who answer questions about prostate cancer treatment within their area of specialization. The library contains books about treatment options and side effects illustrated with graphics, photographs, and animations. The support group room allows patients to listen to the experiences of prostate cancer survivors who have undergone treatment. Groups of three survivors stratified by treatment type are represented through videos, and patients have the option to learn how these survivors have coped with the decision-making process, posttreatment issues, and potential side effects. In addition, the emotional aspects of a prostate cancer diagnosis have been addressed throughout the software. The library books include statements that attempt to normalize the diagnosis, to reduce negative affect and discourage avoidant coping, and to encourage problem-solving coping—for example, “talk about your feelings and concerns, and learn as much as you can about your cancer and treatment.” In the support group room, survivors talk about how they have coped with the disease, treatment decision making, and side effects. Additionally, physicians provide normalizing statements while talking about treatment and side effects (eg, “You are not alone in this diagnosis, thousands of men are being diagnosed with prostate cancer every year”). The detailed developmental and usability testing process has been described elsewhere [11].

### Theoretical Basis of PIES

Self-regulation theory [12,13] guided the development of PIES and the selection of study outcomes. Self-regulation theory postulates two parallel processing arms, one for cognitive and one for affective representations of a health threat or stimulus [12,13]. Cognitive processes are characterized by illness representation attributes such as knowledge or beliefs about a threat or stimulus, its causation, consequence, duration, controllability, and overall understanding (ie, illness cohesion). At the same time, affective processes occur in reaction to the stimulus. The importance of affect in decision-making processes has recently been recognized [14]. Negative affect triggered by the cognitive appraisal of a health threat could in turn influence further information processing and bias decision-making processes [14]. Thus, both cognitive and affective factors influence information processing, decision making, and ultimately behavior, and therefore need to be addressed when providing information to patients.

Research has shown that information processing preferences such as high- versus low-monitoring information-seeking styles also play a role in information processing and decision making [15,16]. A monitoring information-seeking style can be conceptualized as a tendency for individuals to select, encode, interpret, react affectively to, and manage threatening medical health information in either a high- or low-monitoring information-seeking style. A *h* *igh* *-* *monitoring* style is characterized by an increased need for information and by scanning for and magnifying stress-related cues relevant to one’s health, whereas a *low* *-* *monitoring* style is characterized by a reduced need for information, distraction, and minimization of health cues. Previous studies found that monitoring was significantly associated with differential cognitive–affective responses and coping with health-related stressors (eg, a cancer diagnosis) [17-19]. Thus, we explored the influence of text information offered through PIES’ library tailored to a high- or low-information style on information processing and decision variables. We expected that tailored information would improve information processing and facilitate decision making.

The purpose of this study was twofold: (1) to examine the acceptability and feasibility of the PIES intervention and to report preliminary outcomes of the program in a randomized pilot trial among patients with newly diagnosed prostate cancer, and (2) to explore the potential impact of tailoring PIES messages to participants’ information-seeking styles on study outcomes. Following recommendations of assessing feasibility in applied intervention research [18], we evaluated the feasibility of the PIES intervention based on (1) *acceptability of PIES* (ie, successful implementation of the PIES intervention; relevance and acceptability), (2) *r* *ecruitment and r* *etention* (ie, participation and attrition rates), (3) *t* *imeline* (ie, ability to offer the intervention as planned shortly after diagnosis and to assess study outcomes), and (4) *p* *reliminary o* *utcomes* (ie, within the context of a small pilot study, preliminary evidence for the effect of the program on decision-making variables [16]). We hypothesized that patients assigned to use PIES (1) would evaluate the PIES program positively as indicated by high usability ratings, high satisfaction with the information presented, and high ratings of the helpfulness of the program in making a treatment decision, and (2) would be more satisfied with the information they received, be better informed, display less decisional distress, and have more decisional certainty than the men who were assigned to the control condition (ie, received National Cancer Institute [NCI] brochures on prostate cancer); and that (3) men in the tailored PIES group would be more satisfied with the information they received, be better informed, display less decisional distress, and have more decisional certainty than the men in the PIES without tailoring group.

## Methods

### Procedure and Inclusion/Exclusion Criteria

**Figure 1:**
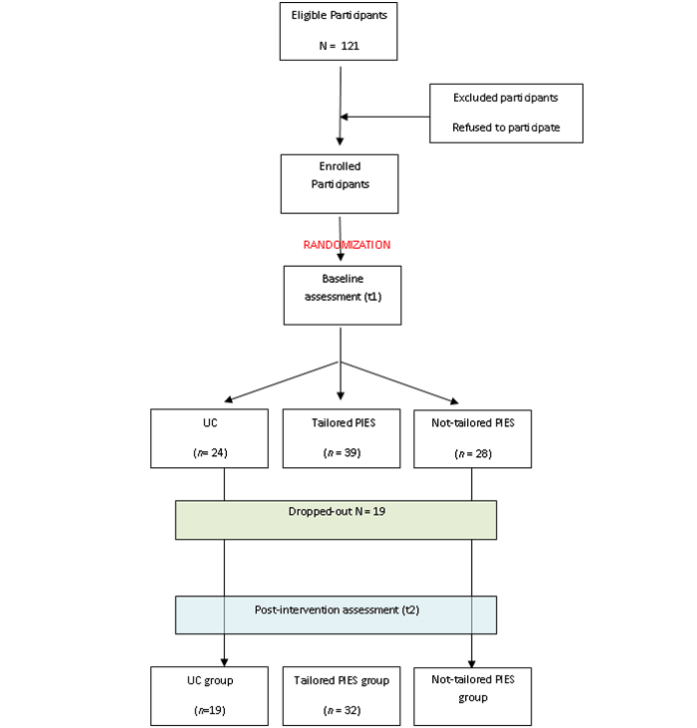
Participants’ flow through the study. PIES = Prostate Interactive Educational System; UC = control group.

We recruited patients with newly diagnosed prostate cancer (T1–2N0M0) who presented themselves at an urban medical center in the Northeast of the United States (n = 121) for a consultation about treatment options between June 2005 and December 2007(see Figure 1). Eligibility criteria were a diagnosis of localized prostate cancer during the past 4 to 6 weeks, ability and willingness to provide written informed consent, and fluency in English. Exclusion criteria were serious comorbidities that would limit patients’ treatment options (eg, cardiovascular diseases that would prohibit a surgical approach). The institutional review board of Mount Sinai School of Medicine reviewed and approved the study.

Collaborating physicians introduced the study to eligible patients and obtained permissions for study personnel to contact patients. If patients agreed to be contacted they were telephoned by the research staff, the study was discussed in detail, and patients were asked to arrive 1 hour prior to their physician appointment. All participants provided written informed consent. To maximize the limited subject pool we randomly assigned patients following a 2:2:1 ratio into 1 of 3 study groups (PIES with tailoring, PIES without tailoring, and the control condition). All data were self-reported and collected through self-administered questionnaires with assistance by the research assistant if necessary (t1 = baseline prior to intervention/comparison conditions; t2 immediately following the intervention/comparison conditions). For tailoring purposes patients in the PIES condition also completed an abbreviated electronic version of the Monitoring/Blunting Style Scale [19]. Based on published cut-offs for monitoring/blunting style the software assigned the patient into 1 of the 2 monitoring/blunting conditions [19].

### Tailoring

The PIES expert system presented all written information available in the library according to the patient’s preferred monitoring style. Specifically, patients in the high-monitoring group received detailed and lengthy descriptions of disease- and treatment-related processes, enhanced by animations and graphics. Descriptions for low monitors, in contrast, were brief, lacking extensive detail, and access to animations and graphics was optional.

### Comparison Condition

We designed the comparison condition as an attention control condition that incorporated elements of usual care. Patients received NCI-published brochures that are routinely provided by physicians: (1) *Understanding Treatment Choices for Prostate Cancer* [20], and (2) *What You Need to Know about Prostate Cancer* [21]. Both booklets explain basic facts about prostate cancer, and its treatment and side effects. All patients were asked to read the brochures for 45 minutes, the identical time patients in the intervention condition had to explore PIES.

### Study Measures

#### Demographic and Clinical Characteristics

The baseline questionnaire included demographic (eg, age, ethnicity, marital status, educational, and employment) information. Self-reported medical variables were verified through a chart review (eg, prostate-specific antigen [PSA] level, time of diagnosis, and treatment preferences).

#### Usability Measures

Guided by previous intervention trials [22] and the recommendations of the Science Panel on Interactive Communication and Health [23], we designed and used 17 items to assess the usability of PIES and the NCI brochures with respect to improving understanding about prostate cancer treatment and its side effects, enhancing treatment decision making, and addressing concerns about the disease, treatment side effects, and cure. Examples of items used are “How useful was the information you received?” “How satisfied are you with the information you received?” “How helpful was the information you received in making a treatment decision?” “Was the information you received confusing?” Responses were endorsed on a 5-point scale (ie, 1 = not al all, to 5 = very much), with higher scores indicating higher levels of satisfaction with the material received.

Additional items to evaluate the usability of PIES were ease of use, clarity, understandability, and helpfulness of the different modules of PIES (eg, library, glossary, and visual materials), resulting in a total of 9 questions. Patients in the 2 PIES groups were asked to endorse on a 5-point scale (1 = not at all; 5 = very much) whether *information was clearly presented* and *easy to understand* (see Table 2 for more examples). Higher scores on these scales represent a more positive evaluation.

### Treatment Decision Measures

We measured decisional variables adopted from the decisional conflict scale [7] with 5 items assessing treatment preferences or decision (“What is your treatment choice?”), confidence in treatment decision (“How confident are you about your treatment choice?”), feeling informed about prostate cancer (“How informed do you feel about prostate cancer?”), need for more information about treatment and side effects (“Would you prefer to have more information to make your treatment decision?”), and need for more time (“Would you prefer to have more time to make your treatment decision?”). Item responses ranged from not at all (1) to very much (5), with higher scores indicating higher levels of decisional confidence, feeling better informed, and an increased need for more time and information. Need for more time and information, confidence in treatment decision, and feeling informed about prostate cancer were measured at postintervention assessment (t2; see Table 4). Treatment preferences were measured at both baseline and postintervention assessments.

### Affect Measure

We used the Impact of Event Scale-Revised (IES-R) [24] to examine psychological distress at baseline (t1). The IES-R is composed of 2 subscales that characterize 2 forms of psychological distress: intrusion (7 items; Cronbach alpha = .84) and avoidance (8 items; Cronbach alpha = .80). Items are measured on a 4-point Likert scale (0–5): not at all (0), rarely (1), sometimes (3), and often (5). The 2 subscales were highly intercorrelated (*r* = .68, *P* < .001). Accordingly, we used an overall mean score to indicate the level of psychological distress, with higher scores indicating higher levels of subjective distress (Cronbach alpha = .88).

### The Monitoring/Blunting Style Scale

We used 8 items of the brief Monitoring/Blunting Style Scale [19] to assess patients’ information-seeking style. The scale consists of 2 scenarios (eg, threat of job loss, going to the dentist), which are followed by 8 potential responses. Of the 8 items of each scenario, 4 describe information seeking or monitoring (eg, “I would talk to my fellow workers to see if they knew anything about the supervisor’s evaluation of me”), and the remaining 4 items describe blunting responses (eg, “I would push all thoughts of being dismissed out of my mind”). A composite score is calculated by subtracting the blunting sum score from the monitoring sum score. High monitoring/low blunting scores (ie, positive scores) indicate a higher tendency toward a monitoring information-seeking style [19]. This measure was administered at baseline assessment (t1) and implemented in PIES for tailoring purposes.

### Statistical Analysis

We analyzed the data with SPSS for Windows, version 16.0 (IBM Corporation, Somers, NY, USA) using descriptive statistics, *t* test, chi-square test, and analysis of variance procedures. Evidence for the acceptability and feasibility of the PIES intervention is demonstrated by (1) high participation and low attrition rates, (2) positive evaluation of PIES, and (3) significant differences between the 2 PIES groups and the comparison control group in postintervention outcomes. Evidence for a significant effect of tailoring the PIES intervention to the patients’ monitoring/blunting styles is demonstrated by significant differences in post-PIES decision-related outcomes between the 2 PIES groups (ie, tailored versus nontailored intervention groups).

## Results

Presentation of results is divided into 4 parts, which describe (1) enrollment and attrition analyses, (2) sample demographic and clinical characteristics, (3) program evaluation, and (4) the impact of PIES on study outcomes.

### Enrollment and Attrition Analyses

Of the 121 referred eligible patients, 91 agreed to participate in the study and completed the baseline questionnaire (t1; 75% acceptance rate). Reasons for nonparticipation were a lack of time and interest, or having made a treatment decision.

To examine any potential bias introduced through selective attrition, we compared patients who completed the study (n = 72) with patients who did not (n = 19) on demographic (eg, age, marital status, race, employment, and education levels), clinical (eg, age, PSA level, treatment preferences), and psychological variables (eg, baseline distress). Almost 80% of patients also completed the t2 assessments (72/91, 79%), providing evidence that the PIES program can be integrated into the clinical counseling routine. The most common reason for not providing the immediate postintervention assessments was lack of time, as patients were called into the doctor’s office for their appointment (ie, “running out of time” and “having a doctor appointment”). Results showed no significant differences between patients who dropped out and patients who completed the postintervention assessment (ie, all *P* > .05).

### Sample Demographic and Clinical Characteristics

The majority of the sample (n = 91) were white (59%), were married (72%), and had a college or higher education (56%); 49% were currently employed. Average PSA level at diagnosis was 7.84 (SD 7.71) µg/L. At baseline patients expressed treatment preferences for brachytherapy (38%), prostatectomy (20%), 3-dimensional conformal radiation therapy (11%), active surveillance (13%), and other treatments (19%, eg, hormone therapy, or complementary and alternative medicine). The majority of patients (87%) had access to a home computer and the Internet (88%); additionally 29% had access to a computer at work. Examining differences between the 3 study groups with regard to baseline demographic, clinical, psychological distress variables, and monitoring/blunting information-seeking style showed no significant differences (see Table 1). Therefore, demographic and clinical variables were not included in comparative analyses as covariates. Subsequent comparative analyses included data only from patients who completed the baseline and post-PIES or control condition assessments.

**Table 1:** Characteristics and decisional outcomes at baseline (t1), by study group

			Total sample (n = 72)	Comparison groups				
				Control condition (n = 19, 26%)	PIES^a^ with tailoring (n = 32, 45%)	PIES without tailoring (n = 21, 29%)	*F*/χ^2^	*P* value
**Demographic and clinical characteristics**								
	Age (years), mean (SD)		61.93 (8.08)	64.16 (8.35)	60.03 (7.77)	62.81 (8.01)	1.76	.18
	PSA^b^ level (µg/L), mean (SD)		7.84 (7.71)	7.69 (3.08)	7.63 (9.45)	8.25 (7.14)	0.08	.09
	Time since diagnosis (weeks), mean (SD)		8.59 (18.88)	9.27 (16.79)	9.32 (123.20)	7.25 (13.58)	10.0	.98
	≤High school, n (%)		30 (44%)	7 (41%)	13 (42%)	10 (50%)	4.50	.60
	≥College, n (%)		38 (56%)	10 (59%)	18 (58%)	10 (50%)		
	Married/with partner, n (%)		51 (72%)	15 (83%)	22 (69%)	14 (67%)	8.40	.39
	Single/widowed/divorced, n (%)		20 (18%)	3 (17%)	10 (31%)	7 (33%)		
	White, n (%)		41 (59%)	10 (56%)	17 (55%)	14 (66%)	5.65	.46
	African American, n (%)		19 (27%)	6 (33%)	8 (26%)	5 (24%)		
	Hispanic/Asian/other, n (%)		10 (14%)	2 (11%)	6 (19%)	2 (10%)		
	Employed, n (%)		34 (49%)	8 (42%)	20 (63%)	6 (29%)	7.26	.12
	Not employed/retired, n (%)		35 (51%)	11 (58%)	10 (37%)	14 (71%)		
**Baseline treatment preferences**								
	Surgery, n (%)		13 (20%)	0 (0%)	8 (29%)	5 26%)	8.4	.40
	External beam radiation therapy, n (%)		7 (11%)	3 (18%)	3 (11%)	1 (5%)		
	Brachytherapy, n (%)		24 (38%)	7 (41%)	9 (32%)	8 (42%)		
	Active surveillance, n (%)		8 (13%)	3 (18%)	2 (7%)	3 (16%)		
	Other, n (%)		12 (19%)	4 (24%)	6 (21%)	2 (11%		
**Baseline psychological covariates**								
	**Psychological distress score**						0.54	.58
		Mean (SD)	1.70 (0.68)	1.88 (1.07)	1.68 (1.03)	1.54 (0.81)		
		Range	0–4	0–4	0–4	0–3		
	**Monitoring style score**						2.09	.13
		Mean (SD)	4.27 (2.06)	4.89 (2.18)	3.75 (1.19)	4.50 (2.22)		
		Range	1–8	0–8	1–8	0–8		
	**Blunting style score**						0.05	.94
		Mean (SD)	2.18 (1.59)	2.11 (1.44)	2.25 (1.86)	2.15 (1.30)		
		Range	0–8	0–6	0–8	0–5		

^a^ Prostate Interactive Educational System.

^b^ Prostate-specific antigen.

### Evaluation of PIES and Print Materials

Patients in both PIES groups and the control group were satisfied with the educational materials they received, and reported that the materials improved their understanding of (1) prostate cancer and its diagnosis, (2) treatment options and side effects (3) follow-up health care and (4) support groups and clinical trials (see Table 2). However, when comparing the 2 groups we found that patients in the control group were significantly more likely to report that the information they received (1) was confusing, (2) was too voluminous, and (3) made them more anxious about their treatment decisions (all *P* < .05; see Table 2). These patients were also significantly less likely to report that the information they received helped them make a treatment decision or was emotionally reassuring.

**Table 2:** Evaluation of program and print materials at postintervention assessment (t2), by study group

	Usability assessment items	Total sample (n = 72)	Study group comparisons				
			Control condition (n = 19, 26%)	PIES^a^ with tailoring (n = 32, 45%)	PIES without tailoring (n = 21, 29%)	*F*_2_	*P* value
**Enhancing treatment information, mean (SD)**							
	Provided information was useful	4.15 (0.09	4.11 (1.05)	4.25 (0.67)	4.05 (1.05)	0.35	.71
	Provided information was satisfactory	4.15 (0.08)	3.95 (1.08)	4.25 (0.67)	4.20 (0.77)	0.84	.43
	Provided information was confusing	1.48 (0.84)	1.84 (1.12)	1.25 (0.57)	1.50 (0.82)	3.31	.05
	Provided information was too much	1.63 (1.06)	2.47 (1.22)	1.47 (0.98)	1.10 (0.31)	11.61	.01
	I now understand the prostate cancer diagnosis	3.81 (1.10)	3.84 (1.16)	3.90 (0.94)	3.63 (1.30)	0.34	.62
	I now understand prostate cancer treatment	3.59 (1.22)	3.84 (1.17)	3.57 (1.23)	3.37 (1.30)	0.70	.50
	I now understand prostate cancer side effects	3.89 (1.01)	3.84 (1.07)	3.90 (0.98)	3.90 (1.04)	0.03	.98
	I now understand follow-up care	3.78 (0.99)	3.78 (1.11)	3.87 (0.97)	3.63 (0.96)	0.32	.93
	I now understand clinical trials	3.23 (1.24)	3.47 (1.42)	3.27 (1.19)	2.80 (1.11)	1.24	.30
	Made me consider more questions to ask	3.73 (0.91)	3.74 (0.81)	3.72 (0.89)	3.75 (1.07)	0.01	.99
	Made me seek more information about prostate cancer	3.45 (1.0)	3.63 (0.93)	3.41 (1.07)	3.35 (0.90)	0.45	.95
**Enhancing decision making** **, mean (SD)**							2
	The information is helpful in decision making	3.56 (1.38)	1.79 (0.92)	4.29 (0.64)	4.10 (1.07)	55.62	.01
	Made me think about my treatment choices	3.90 (0.88)	3.89 (0.81)	3.88 (0.7)	3.95 (1.10)	0.04	.96
	Made it difficult for me to decide	2.46 (0.91)	2.68 (1.11)	2.39 (0.92)	2.35 (0.93)	0.71	.94
	Calmed my nerves about my decision	3.10 (1.94)	2.68 (1.06)	3.12 (0.83)	3.46 (0.89)	3.46	.04
	Made me more anxious about my decision	2.66 (1.18)	3.62 (1.05)	2.45 (1.09)	2.40 (1.27)	3.74	.03
	Made the treatment options clear for me	3.58 (0.68)	3.28 (1.27)	3.72 (0.73)	3.63 (0.76)	1.75	.22

^a^ Prostate Interactive Educational System.

### Differences Between the Tailored and Nontailored PIES Program Evaluation

We found no differences between the tailored and nontailored PIES group among the following group of variables: demographic and clinical factors, PIES evaluation and decision variables, and monitoring style (see Table 3). Thus, data of these 2 groups were combined in subsequent analyses and compared with data from the comparison group (ie, control condition).

**Table 3:** Prostate Interactive Educational System (PIES)-specific evaluation of the library materials at postintervention assessment (t2) among tailored and nontailored PIES groups^a^

Usability assessment items	Study group comparisons			
	PIES with tailoring (n = 32)	PIES without tailoring (n = 21)	*t*_42_	*P* value
PIES information in library is clearly presented	3.77 (0.97)	3.75 (0.80)	0.29	.77
PIES includes everything I need to know	3.67 (1.09)	3.25 (0.91)	1.18	.25
Information is more than I want to know	2.53 (1.01)	2.60 (0.88)	0.35	.73
Graphics are clear	4.03 (0.88)	4.05 (0.69)	0.31	.76
Glossary is helpful	3.93 (0.93)	3.80 (0.73)	0.41	.68
Library was easy to understand	3.96 (1.00)	3.63 (0.82)	1.50	.14
Library provided all the information I need	3.56 (1.09)	3.32 (1.27)	0.60	.55
Library helped me with the decision	3.48 (0.96)	4.47 (0.77)	0.13	.09
Library has more information than what I want	2.68 (1.06)	2.58 (0.96)	0.22	.82

^a^ Data are mean (SD).

### Examining Preliminary Outcomes: Decisional Variables

We compared decisional process variables between the combined PIES groups and the control group immediately following the intervention (ie, at t2). Results indicated that patients in the PIES groups were significantly more confident about their treatment preferences and significantly less likely to report that they needed more information to make a decision. Moreover, patients in the PIES group indicated that they would need less time to deliberate their treatment options and felt better informed about their choices and potential side effects (see Table 4). Despite these significant differences in decisional process variables, treatment preferences remained stable between the PIES and control group, and no significant impact of PIES on treatment preferences was found (see Table 4).

**Table 4:** Decisional outcomes at postintervention assessment (t2), by study group

Treatment preferences/decision assessment items		Total sample (n = 72)	Study group comparisons				
			Control condition (n = 19, 26%)	PIES^a^ with/without tailoring (n = 53, 74%)	*t*/χ^2^	Degrees of freedom	*P* value
**Decisional variables at t2, mean (SD)**							
	Have confidence in treatment decision made	3.69 (1.10)	3.22 (1.32)	3.85 (1.022)	–2.35	68	.02
	Preferred more time to think about options	2.52 (1.41)	3.00 (1.41)	2.33 (1.42)	1.81	68	.07
	Preferred more information about prostate cancer	2.77 (1.5)	3.44 (1.54)	2.52 (1.49)	2.48	68	.02
	Feeling informed about prostate cancer and treatment	3.57 (0.91)	3.28 (1.07)	3.74 (0.089)	–1.65	68	.10
**Treatment preference at t2** **, n/N (%)**							
	Surgery	14/65 (22%)	0/16 (0%)	14/49 (29%)	7.3	4	.11
	External beam radiation therapy	11/65 (17%)	5/16 (31%)	6/49 (12%)			
	Brachytherapy	28/65 (43%)	8/16 (50%)	20/49 (41%)			
	Watchful waiting/active surveillance	4/65 (6%)	1/16 (6%)	3/49 (6%)			
	Other	8/65 (12%)	2/16 (13%)	6/49 (12%)			

^a^ Prostate Interactive Educational System.

## Discussion

Making a treatment decision under conditions of heightened uncertainty, due to the absence of an evidence-based criterion standard treatment option, is difficult and may lead to increased distress, difficulty making a treatment choice, and feelings of decisional regret, especially when treatment outcomes decrease patients’ quality of life. Involving prostate cancer patients in treatment decision making has been repeatedly advocated [25]; however, evidence has shown that patients often have difficulties processing treatment-related information, especially in an emotionally charged situation following a cancer diagnosis. We designed the PIES program to address this issue. Our findings showed that patients in the PIES groups, compared with those in the control group were significantly more likely to report higher levels of confidence in their treatment choices, to rate the helpfulness of the information significantly higher for making a treatment decision, and to indicate that the information was emotionally reassuring. Additionally, the participants in the PIES groups thought the information was clear and understandable, were significantly less likely to report a need for more information, and were less anxious about their treatment choices.

### Enrollment and Attrition Analyses

Following previous research assessing the feasibility of an applied intervention [18,25], we examined (1) *recruitment* and *retention*, (2) the *acceptability* of PIES, and (3) *preliminary outcomes*. The high acceptance and completion rate of the baseline questionnaire (80%) suggested that patients participating in the study had a chance to explore the PIES program and found it acceptable.

### Evaluation of PIES and Print Materials

Our study design included a time and attention comparison condition, the methodologically most rigorous approach to test a novel intervention. Patients in the control condition were asked to read 2 NCI-published brochures that provide extensive, albeit noninteractive, information about prostate cancer and its treatment. The comprehensive nature of the provided information might be responsible for the overall increase in understanding among patients in the control group. Despite a uniform increase in being informed, patients completing PIES were significantly less confused about their treatment options, and felt that the information they received was the “right” amount and was emotionally reassuring. The latter point is particularly noteworthy, as it underscores the importance of addressing the inherently distressing nature of a prostate cancer diagnosis. Guided by our theoretical self-regulation framework that incorporates both cognitive and emotional processing of health-relevant information, we addressed the emotional aspect of prostate cancer throughout the PIES program. Survivors talked freely about the emotional impact of prostate cancer on themselves and their family, and how they coped with the diagnosis. Physicians attempted to emotionally reassure patients by providing normalizing statements (eg, “you are not alone in this diagnosis, thousands of men are being diagnosed with prostate cancer”). They also made references to available support from psychologists and social workers, and discussed medical solutions to erectile and urinary dysfunction. Although the program needs to be evaluated further to relate specific components of PIES usage to specific decision and adjustment variables, the overall results support our comprehensive cognitive–affective approach to patient information.

### Differences Between the Tailored and Nontailored PIES Program Evaluation

The lack of an effect of the tailoring variable was surprising. There are two potential reasons for this outcome. First, we only tailored written information contained in the library books; all physician answers and patient stories were nontailored. As patients were free to explore the program at will, some might have spent more time with patient stories and physician answers, rather than reading the tailored written information contained in the library. Second, patients might have self-tailored their information intake by acquiring the amount and detail of information that corresponded to their monitoring style. As exploration of the software was unstructured, it is reasonable to assume that they would explore those issues that were relevant and interesting to them and avoid those items that might be uninteresting or anxiety provoking. It is therefore possible that patients’ self-tailoring exploration behavior superseded the tailoring capabilities of the software, particularly if patients did not spend enough time in the library reading the tailored materials. Although preliminary results from our study suggest that patients might engage in self-tailoring activities, the issue of tailoring to information processing within a multimedia environment needs to be examined in more detail before a comprehensive recommendation about tailoring to this variable can be made. In addition to demonstrating acceptability on the patient side, we further demonstrated that implementation into a clinical consultation service is possible. Despite some time constraints that did not allow all patients to complete the postintervention questionnaire, a large majority completed all assessments. This problem could be mitigated in future implementations by making the program available to patients via the Internet prior to their physician appointment. It would be simple to provide patients with the program’s Internet link and an individualized login and password to access the software, when they make an appointment to see the physician. The next step then would be to link the program with the electronic medical record system that could transmit patients’ concerns and preferences to the treating physician prior to the consultation. This would give physicians valuable information about the upcoming consultation, and would allow them to tailor the information provided more closely to patients’ needs.

### Preliminary Outcomes: Decisional Variables

The results of the study indicate that PIES improved some of the decisional process variables under study. Specifically, PIES succeeded in satisfying the patients’ information needs, increased their confidence about their treatment preferences, and provided the information in an emotionally reassuring way. PIES also increased patients’ knowledge about their treatment options and associated side effects. These results are particularly promising given that they were obtained within the framework of a time and attention comparison condition (ie, control group), which provided considerably more information than standard or usual care. Patients receiving standard or usual care might or might not receive written information to take home in addition to the physician consultation. In the present study patients received 2 brochures and were asked to spend 45 minutes reading both of them; thus, they were most likely better informed than the average patient attending a physician consultation. We would expect, therefore, that PIES would perform even better when compared with standard or usual care.

As expected, PIES did not change patients’ treatment choice. Because PIES was designed with the intention to inform patients and to help them identify what is important to them with regard to treatment outcomes and future quality of life, the lack of a significant effect on change in treatment choices was not unexpected and indeed affirmed our approach.

No study is without limitations and the present one is no exception. The study findings confirmed PIES’ acceptability and demonstrated its ability to influence important decisional variables. Although the results are not definitive, documenting acceptability and feasibility and preliminary results of its effect is an important first step before proceeding to a larger randomized controlled trial. Second, our patient population was not representative of the general population, as the majority were well educated (56% college educated or higher). Third, we examined study outcomes before and after the completion of PIES intervention. Examining the impact of the intervention several weeks later might reveal a stronger impact of the intervention on psychosocial outcomes such as regret, recurrence worries, and psychological stress.

In sum, the present study provides evidence that PIES is acceptable to patients, can be implemented into a routine clinic program, successfully improves patients’ knowledge about treatments and side effects, and increases their confidence in treatment decision making.

## Multimedia Appendix 1

The virtual health center.

## Multimedia Appendix 2

The Library.

## Multimedia Appendix 3

The physicians’ offices.

## Multimedia Appendix 4

The support group room.

## Abbreviations

IES-R: Impact of Event Scale-Revised
NCI: National Cancer Institute
PIES: Prostate Interactive Educational System
PSA: prostate-specific antigen
