# Supplementary figures and images for: Acceptability and Preliminary Feasibility of an Internet/CD-ROM-Based Education and Decision Program for Early-Stage Prostate Cancer Patients: Randomized Pilot Study
Source: J Med Internet Res. 2012 Jan 13;14(1):e6. doi: 10.2196/jmir.1891 (PMC3846339; doi:10.2196/jmir.1891)

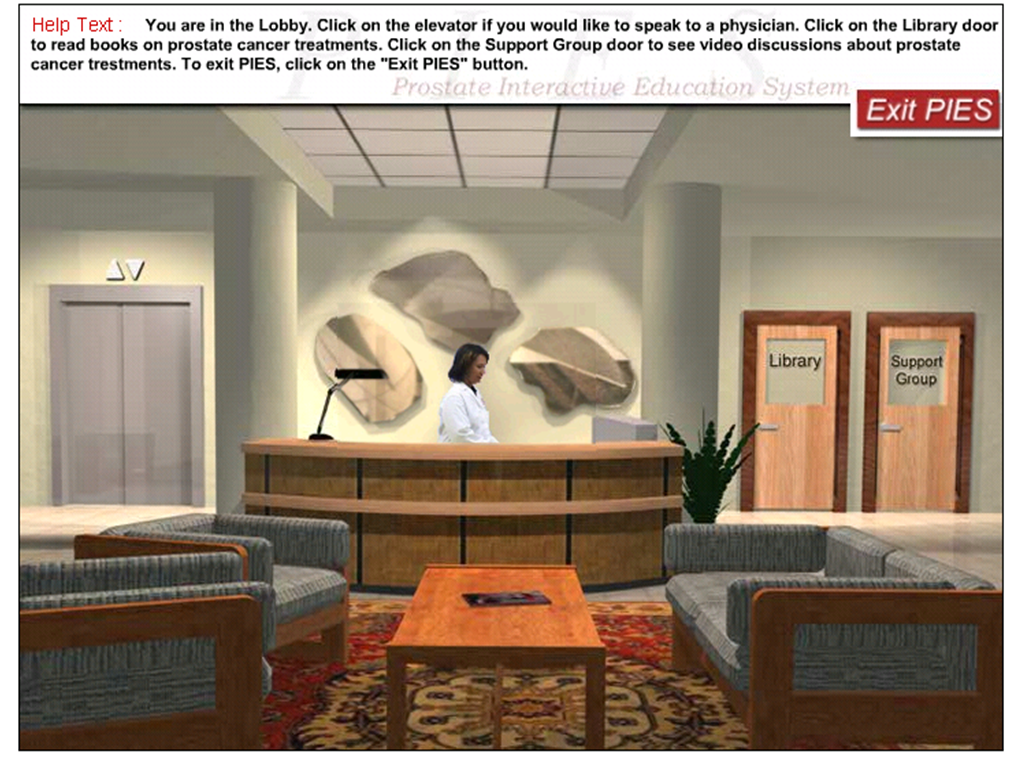

Supplement: Supplementary file 1 [file jmir_v14i1e6_app1.png]

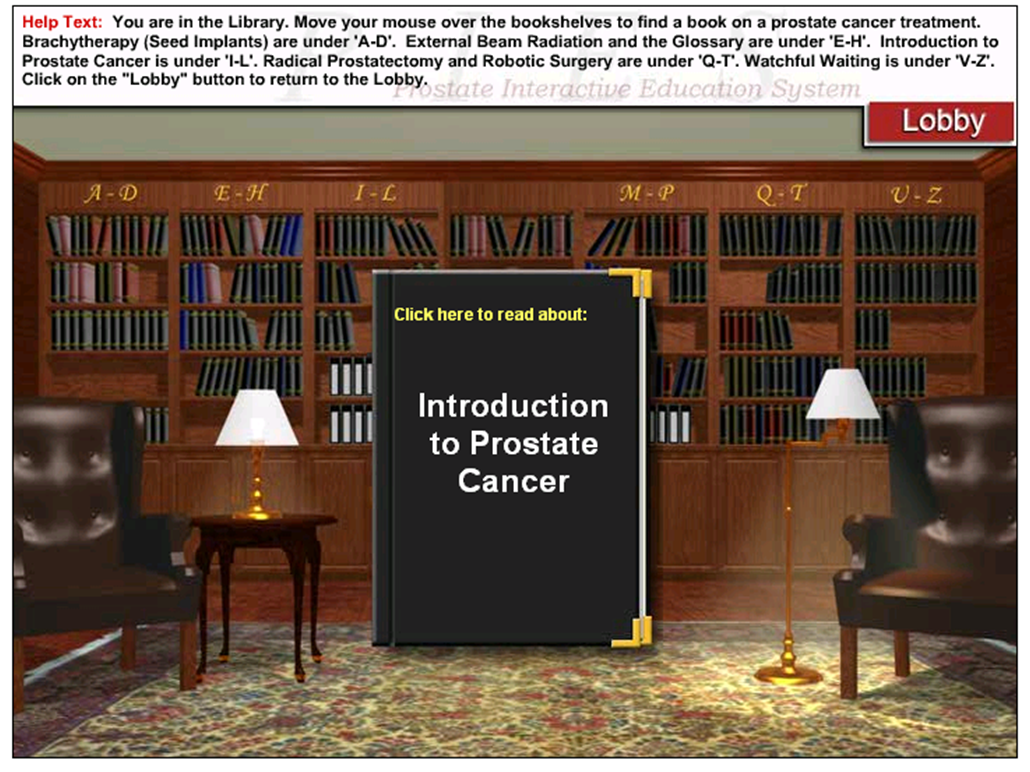

Supplement: Supplementary file 2 [file jmir_v14i1e6_app2.png]

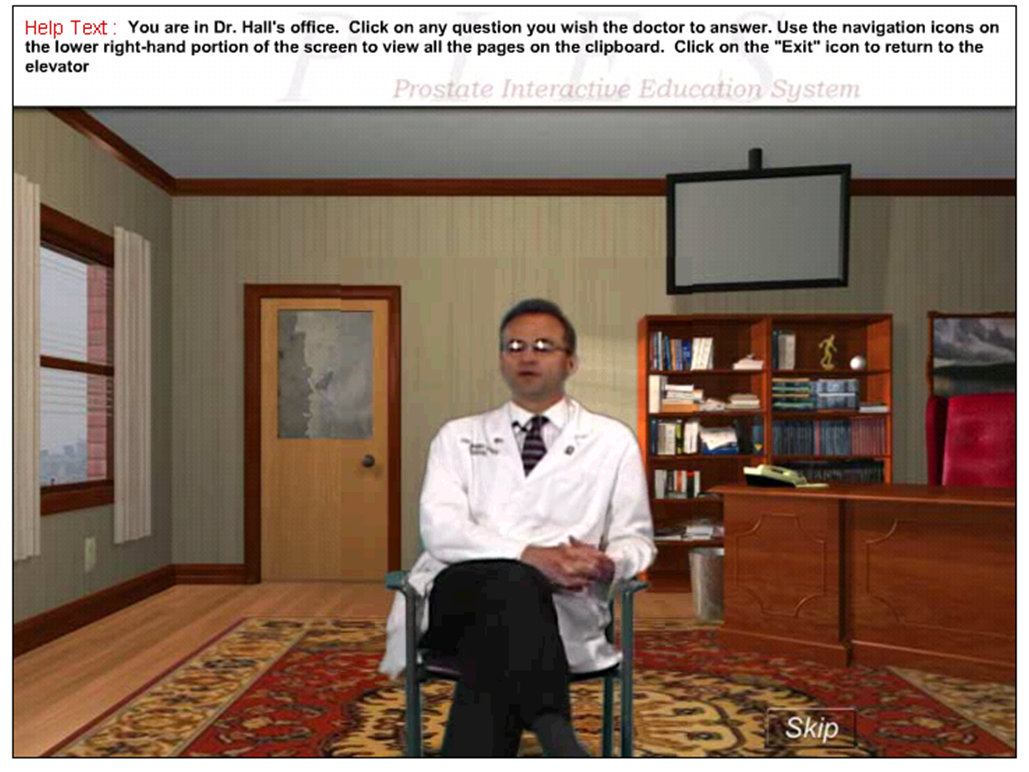

Supplement: Supplementary file 3 [file jmir_v14i1e6_app3.png]

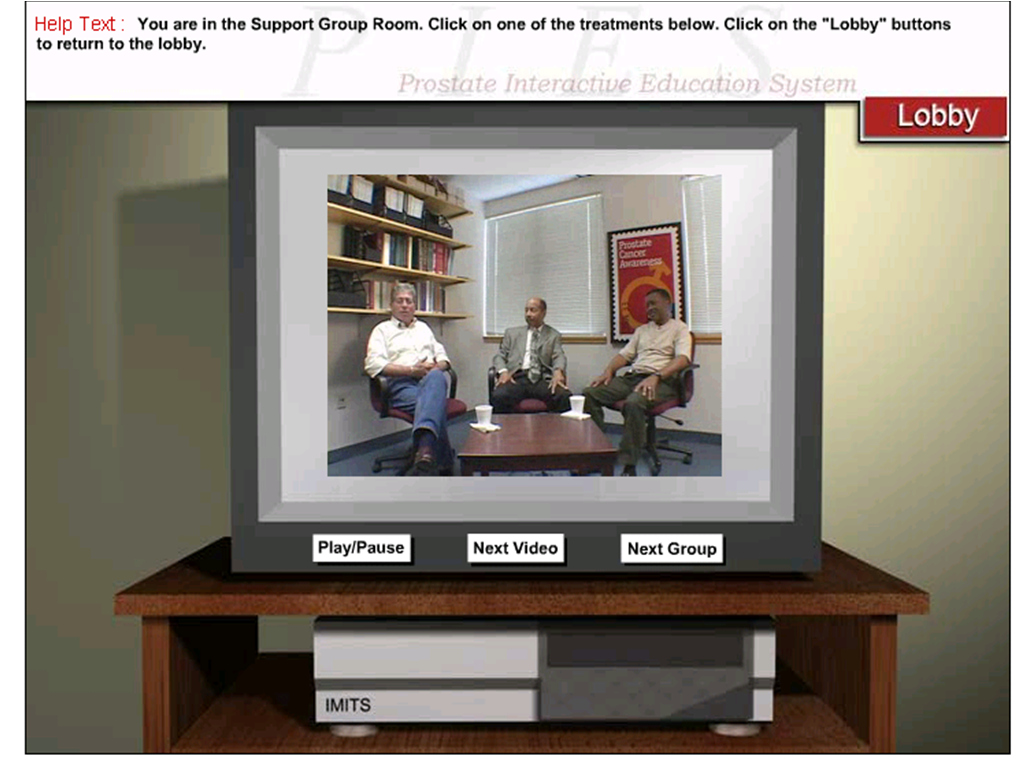

Supplement: Supplementary file 4 [file jmir_v14i1e6_app4.png]
